# Multidrug resistance transporter profile reveals MDR3 as a marker for stratification of blastemal Wilms tumour patients

**DOI:** 10.18632/oncotarget.14491

**Published:** 2017-01-04

**Authors:** Lourdes Hontecillas-Prieto, Daniel J. Garcia-Dominguez, Diego Pascual Vaca, Rosa Garcia-Mejias, David Marcilla, Gema L. Ramirez-Villar, Carmen Saez, Enrique de Álava

**Affiliations:** ^1^ Institute of Biomedicine of Seville (IBiS), Hospital Universitario Virgen del Rocío/CSIC/Universidad de Sevilla, Seville, Spain; ^2^ Pathology Unit, Hospital Universitario Virgen del Rocío/CSIC/Universidad de Sevilla, Seville, Spain; ^3^ Pediatric Oncology Unit, Hospital Universitario Virgen del Rocío/CSIC/Universidad de Sevilla, Seville, Spain

**Keywords:** Wilms tumours, multidrug resistance transporters, MDR3, MRP1, blastemal stratification

## Abstract

Wilms tumour (WT) is the most common renal tumour in children. Most WT patients respond to chemotherapy, but subsets of tumours develop resistance to chemotherapeutic agents, which is a major obstacle in their successful treatment. Multidrug resistance transporters play a crucial role in the development of resistance in cancer due to the efflux of anticancer agents out of cells. The aim of this study was to explore several human multidrug resistance transporters in 46 WT and 40 non-neoplastic control tissues (normal kidney) from patients selected after chemotherapy treatment SIOP 93–01, SIOP 2001. Our data showed that the majority of the studied multidrug resistance transporters were downregulated or unchanged between tumours and control tissues. However, *BCRP1*, *MDR3* and *MRP1* were upregulated in tumours versus control tissues. *MDR3* and *MRP1* overexpression correlated with high-risk tumours (SIOP classification) (*p* = 0.0022 and *p* < 0.0001, respectively) and the time of disease-free survival was significantly shorter in patients with high transcript levels of MDR3 (*p* = 0.0359). MDR3 and MRP1 play a role in drug resistance in WT treatment, probably by alteration of an unspecific drug excretion system. Besides, within the blastemal subtype, we observed patients with low MDR3 expression were significantly associated with a better outcome than patients with high MDR3 expression. We could define two types of blastemal WT associated with different disease outcomes, enabling the stratification of blastemal WT patients based on the expression levels of the multidrug resistance transporter MDR3.

## INTRODUCTION

Wilms tumour (WT), also called nephroblastoma, is an embryonal malignant neoplasm of the kidney accounting for 6–7% of all childhood cancers [1–3]. WT is the most common renal tumour in children before the age of five years [1, 4–6]. Histologically, the usual appearance of WT is that of a mixed pattern, with variable proportions of three cellular components (blastemal, epithelial, and stromal). Each one of these cellular elements may show different degrees of differentiation [7].

Children's Oncology Group (COG) and the International Society of Paediatric Oncology (SIOP) are the two major groups which have great contributions in the management of WT. Both develop two different approaches or protocols of the diagnosis and treatment of WT [6, 8–9]. Those two protocols are currently used: The COG in North America develops a system based in an upfront surgery (nephrectomy) to accurately assess tumour stage and histology [3, 10]. In Europe, SIOP delays nephrectomy 4–6 weeks favoring upfront chemotherapy and reducing complications of surgery and tumour spillage [3, 11]. Both collaborative groups have proven valuable in predicting outcomes and the different approaches of treatment have shown almost equivalent clinical outcomes [10, 12–13].

Three risk groups (low, intermediate, and high) were defined by the SIOP Pathology system (SIOP 93–01, SIOP 2001), based on the percentage of overall necrosis and the predominant cell type in the residual viable cells [14–15]. Although in most cases of WT such therapy induces a considerable degree of tumour shrinkage and the overall survival is greater than 90%, a proportion of cases show a limited volume reduction and retain an abundance of blastemal tissue post-chemotherapy. These are stratified as high-risk blastemal cases and have a poor prognosis [16]. One of the biggest problems for patients with poor prognosis is resistance to standard chemotherapy [17]. Patients with blastemal WT represent a chemoresistant cohort requiring more intensive adjuvant treatment [18].

Several mechanisms have been described that could contribute to chemoresistance: Nonspecific expulsion of drugs out of the cell is probably the best studied resistance mechanism and mainly related to the action of certain proteins [19–20]. These proteins are present in many normal tissues of the human body as well as in tumours. The fact that many of these have secretory-excretory tissue functions (kidney, liver, gastrointestinal epithelium and respiratory epithelium) suggests that multidrug resistance proteins could play the physiological function of protection against exogenous toxins [21–23]. Over the years, a number of genes have been identified as being involved in multidrug resistance but the molecular mechanisms of drug resistance in WT remain poorly understood. The multidrug resistance transporters are classified into two groups: A) Proteins belonging to the family of transporters known as ABC transporters (ATP-binding cassette), which are located in the cell membrane. Members of this family are involved in the active transport of many molecules through the cell membrane. Some of the main members are P-glycoprotein (PGP), multidrug resistance-associated protein (MRP) and *breast cancer resistance protein* (BCRP), which act as “efflux pump” decreasing the intracellular accumulation of different substances, including many cytostatic drugs [24–25]. B) Non-ABC transporters such as related protein in lung resistance (LRP) or Major Vault Protein (MVP): Vault human complex comprises the vault higher molecular weight protein (MVP) and two minor vault proteins (VPARP and TEP1), plus untranslated RNA molecules. Vault proteins are found in the cytoplasm, probably associated with cytoplasmic vesicles, and a small part in the nuclear membrane, particularly in the nuclear pore complex [26–28].

The role of multidrug resistance transporters in WT is not well characterized. Since 1997, few studies have addressed the expression of multidrug resistance transporters in this prevalent tumour. These manuscripts provided data on the main transporters but did not provide a complete expression profile. Moreover, the studies showed a reduced number of cases, a lack of suitable statistical analysis and contradictory results [26, 29–33]. It is important to elucidate the role of multidrug resistance transports in WT as they may form the basis of one of the possible reasons for a WT patient's treatment failure. The aim of the present study was to analyze the expression profiling of multidrug resistance transporters in frozen and in paraffin-embedded samples from WT patients and correlate these results with different clinicopathological parameters.

## RESULTS

### Differences in the gene expression profile of multidrug resistance genes between control kidneys and Wilms tumour samples

**Figure 1:**
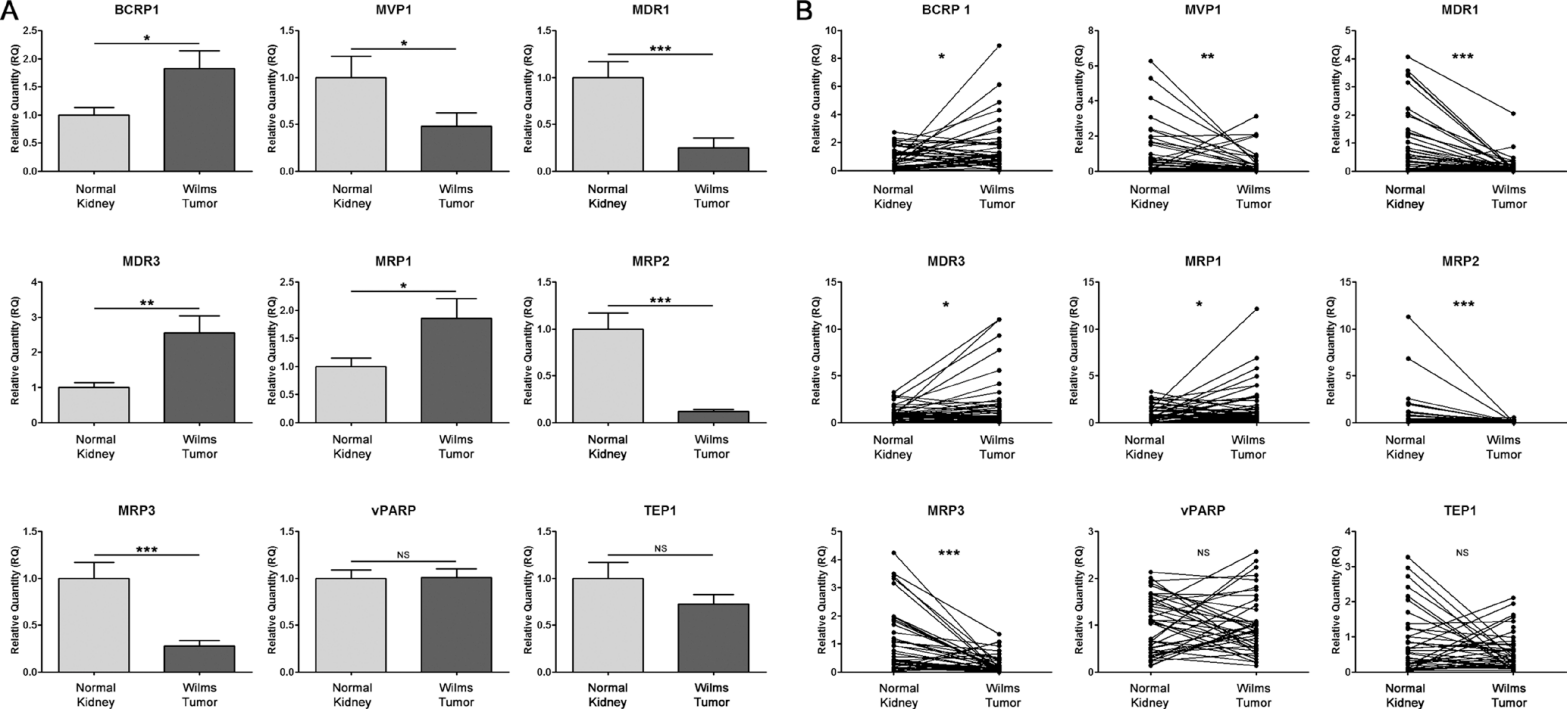
Profiling of the transcript expression of multidrug resistance genes from frozen tissue samples (normal kidney and WT tissues) reveals changes of multidrug resistant gene expression in WT samples (**A**) Analysis of expression in 40 control kidney and 46 WT samples. (**B**) Paired analysis between kidneys (*n* = 40) and WT (*n* = 40). For all the analyses, *p < 0.05; **p < 0.01; ***p < 0.001. NS, not significant.

To determine the role of multidrug resistance transporters in WT, we performed gene expression profiling of nine multidrug resistance transporters in WT frozen samples (Series 1). Previous to qRT-PCR analysis, we assessed the best endogenous gene by geNorm v3 software and evaluated the most stable reference genes from a set of tested genes in a given cDNA sample panel (kidney and WT samples). A gene expression normalization factor was calculated for each tissue sample based on the geometric mean of a user-defined number of reference genes: *B2M, GAPDH, HRPT1* and *TPT1*. The best gene for expression normalization was *TPT1*. Then, through qRT-PCR, levels of mRNAs encoding transporters in WT were compared to those of normal kidneys. We found that expression of *BCRP1* (*breast cancer resistance protein*)*, MDR3* (Multidrug resistance 3) and *MRP1* (Multidrug resistance-associated protein 1) were significantly higher in WT patient samples than in control samples. We also observed a significantly lower expression of *MRP2, MRP3* (Multidrug resistance-associated protein 2 and 3) and *MVP1* in WT than in control samples (Figure 1A). Next, we analyzed the mRNA levels of these genes in paired samples (*n* = 40) and compared its expression between normal kidney and WT (Figure 1B). We observed the same expression pattern as for the non-paired samples. However, we did not find any differences in the minor vault genes (*vPARP* and *TEP1*) in any of the analyses done. Therefore, in WT samples we observed a differential expression pattern in the transporter genes *BCRP1, MDR3* and *MRP1*, which could confer resistance to chemotherapy treatment.

### Overexpression of *MDR3* and *MRP1* correlated with blastemal subtype and high-risk prognosis of Wilms tumour patients

**Figure 2:**
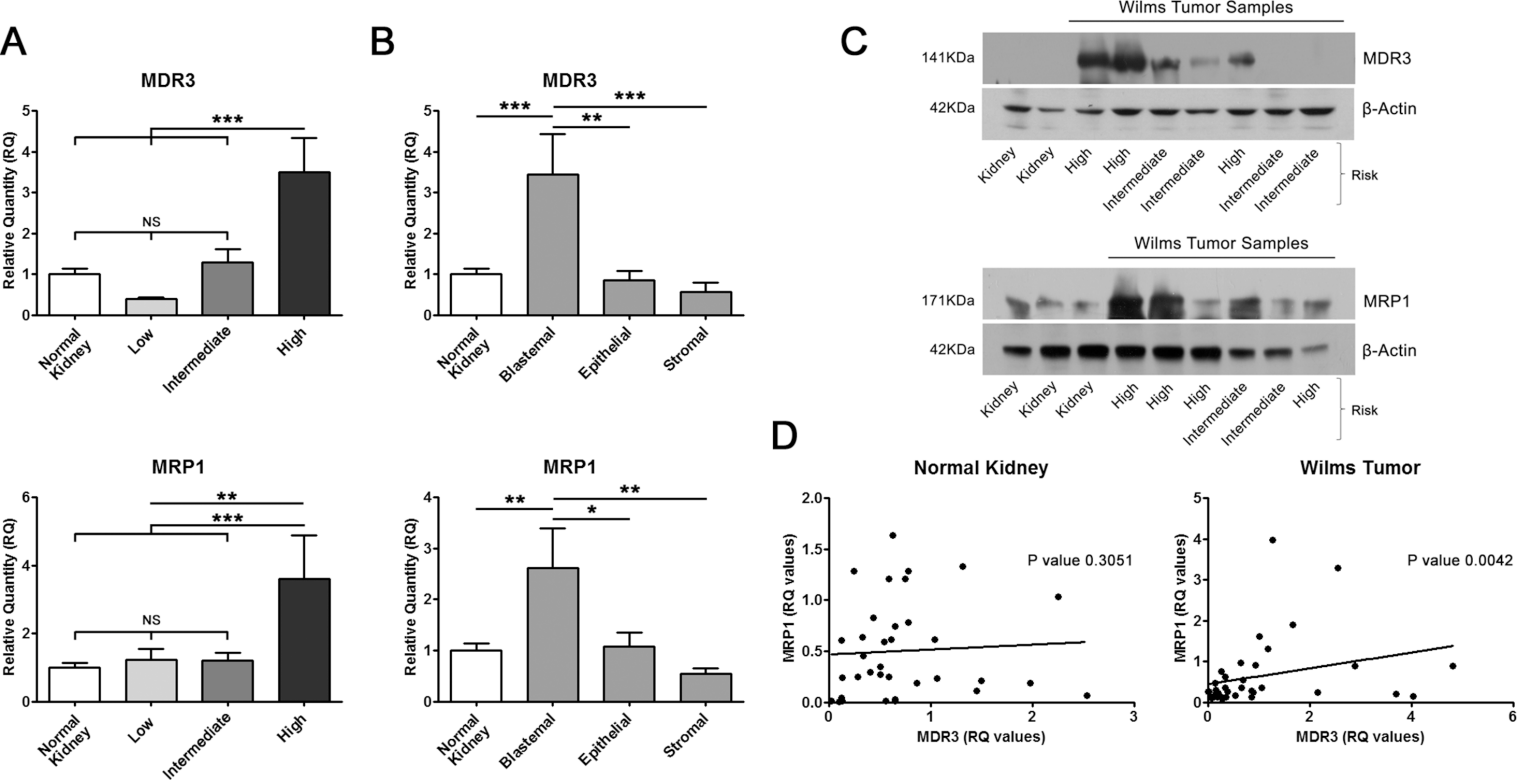
Overexpression of *MDR3* and *MRP1* in tumour samples correlated with poor prognosis (**A**) Significantly higher expression of *MDR3* and *MRP1* in high-risk WT than normal kidney, low and intermediate risk tumours. (**B**) Blastemal predominant tumoural subtype presented more *MDR3* and *MRP1* expression levels than kidney, low- and intermediate-risk tumours. (**C**) Protein expression of MDR3 and MRP1 in normal kidney and WT samples. High-risk WT samples had more MDR3 and MRP1 protein expression than kidneys and intermediate risk tumours. (**D**) Significant positive correlation between *MDR3* and *MRP1* in WT but not in normal kidney samples. For all the analyses, *p < 0.05; **p < 0.01; ***p < 0.001. NS, not significant.

The determined pattern of gene expression provides information on which transporter genes have increased expression in WT (and may confer the tumour more resistance to treatment) and those that have a decreased expression (the tumour would be more sensitive to treatment). Consequently, we compared the tumour expression of multidrug resistance transporter genes with WT clinicopathological characteristics. We analyzed the risk (low, intermediate and high), stage (I to IV), and tumoural subtype (blastemal, epithelial and stromal) of 46 WT samples (Series 1). We observed that only *MDR3* and *MRP1* overexpression showed a significant relationship with clinicopathological parameters. The other multidrug resistance transporters in the series did not correlate with those parameters (data not shown). Surprisingly, *MDR3* and *MRP1* expression showed a significant increase in high-risk tumours compared with low or intermediate risk. Also, we appreciated a progressive increase in *MDR3* expression as the degree of risk was increased (Figure 2A). On the other hand, blastemal tumours showed a stronger expression in *MDR3* and *MRP1* than epithelial or stromal WT (Figure 2B). Subsequently, we checked whether we could detect the same differences in MDR3 and MRP1 protein levels between kidney and tumour samples and between high and intermediate risk WT samples. We extracted protein from 5 kidneys and 13 WT frozen samples and analyzed the expression by western blot (Series 1). We observed that high-risk WT samples had more MDR3 and MRP1 expression than normal kidneys and intermediate-risk tumours (Figure 2C). Therefore, MDR3 and MRP1 transcript and protein levels were increased in high-risk tumours. We were interested to evaluate whether the expression of these two multidrug resistance transporters was correlated. Our results show that in normal tissue samples there was no correlation in expression between the two genes, but in WT samples there was a positive correlation (Figure 2D). So, MDR3 and MRP1 were increased in high-risk tumours, particularly in blastemal tumours, and there was a positive correlation between them. This result indicates that the multidrug resistance transporters MDR3 and MRP1 could be determinant of treatment resistance of malignant WT patients.

### Immunohistochemical detection confirms MDR3 and MRP1 overexpression in Wilms high-risk tumours

**Figure 3:**
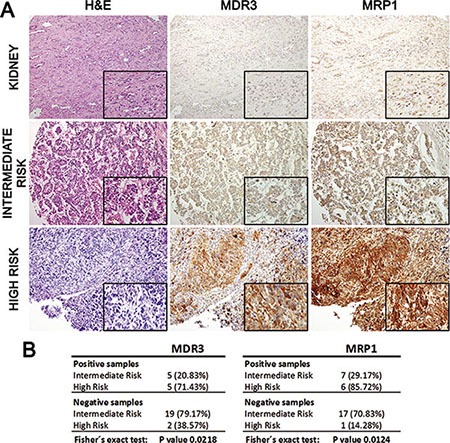
MDR3 and MRP1 immunohistochemical analysis expression in 31 WT patients from Tissue MicroArray (**A**) Immunohistochemical detection of MDR3 and MRP1 in kidney and intermediate and high-risk WT (20× and 40× amplification). High-risk tumours showed a stronger expression of MDR3 and MRP1 than kidney and intermediate-risk tumours. (**B**) Significant correlation between negative and positive expression of MDR3 and MRP1 in intermediate- and high-risk WT samples.

A total of 31 formalin-fixed paraffin-embedded FFPE samples (Series 2) were analyzed using immunohistochemical detection with selected antibodies. We analyzed the presence/absence of MDR3 and MRP1 expression in WT. Similar to the analysis of mRNA levels and in total protein extracts (Figures 1 and 2), we observed a differential expression between kidneys and WT as well as between intermediate- and high-risk tumours. All of the normal kidney samples either expressed weakly or were negative for MDR3, while MRP1 expression was weak to moderate. The high-risk WT samples had higher expression than normal kidney or intermediate-risk WT samples (Figure 3A). Besides an evaluation of the levels of expression, the use of immunohistochemistry allowed us to distinguish the subcellular localization of the two multidrug resistance transporters. We observed plasma membrane and cytoplasmic expression for both transporters. By Fisher´s exact test we confirmed a statistically significant difference in expression in high-risk expression tumours. There was MDR3 expression in 71.43% of high-risk tumours compared with only 20.83% of intermediate-risk tumours. Likewise, MPR1 expression in high-risk tumours was 85.72% with respect to 29.17% in intermediate-risk tumours (Figure 3B). These significant differences confirm the relevance of MDR3 and MRP1 in high-risk WT.

### High expression of *MDR3* correlated with reduced disease-free survival in WT patients

**Figure 4:**
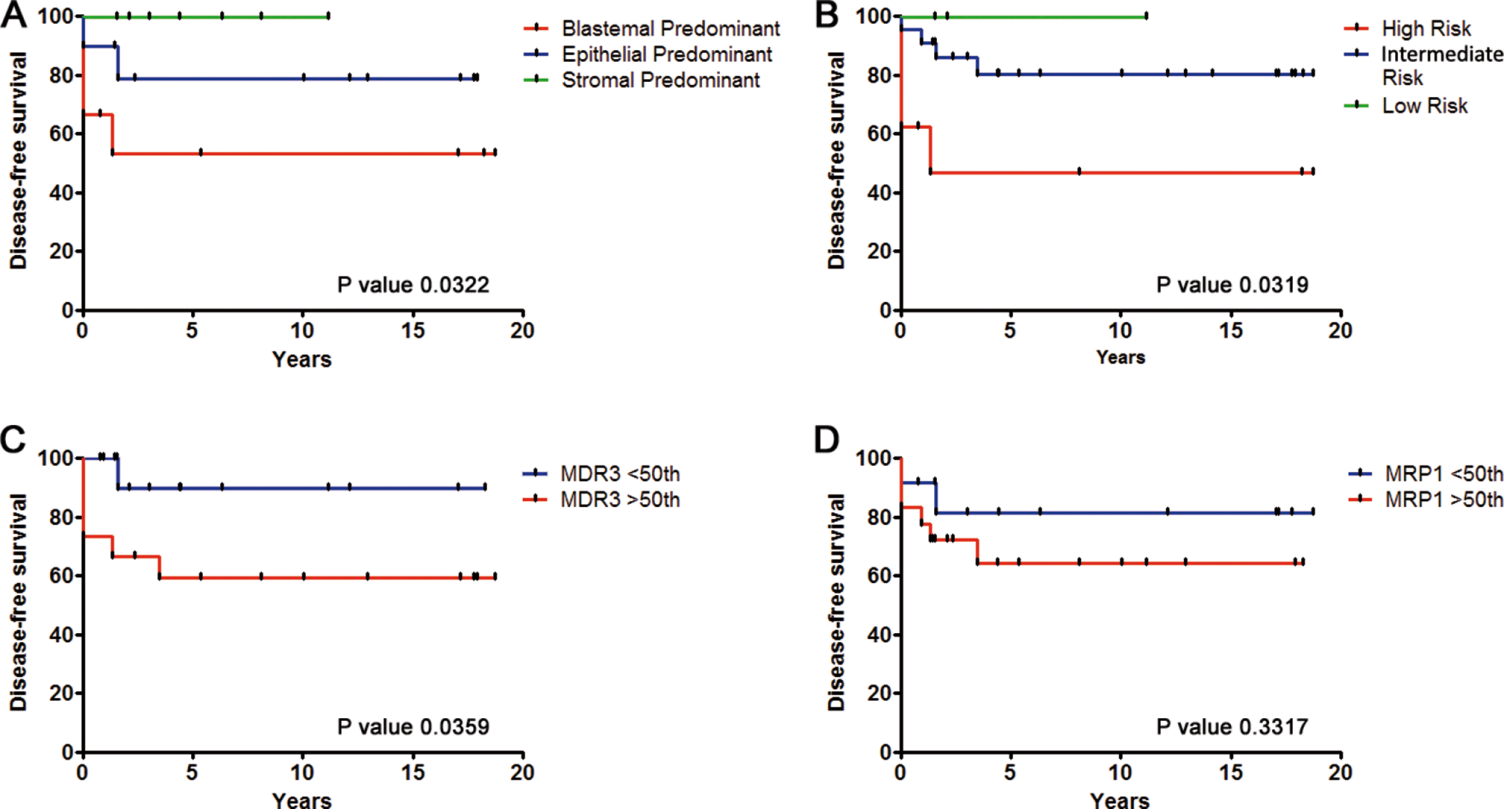
Overexpression of *MDR3* gene is associated with reduced disease-free survival of WT patients (**A**) Blastemal predominant subtype conferred a reduced period of disease-free survival compared to the Epithelial and Stromal subtypes. (**B**) Tumoural risk Kaplan-Meier showed reduced disease-free survival in patients with high-risk tumours. (**C**) Disease-free survival of patients with high *MDR3* expression was significantly shorter than those with low *MDR3* expression. (**D**) *MRP1* Kaplan-Meier disease-free survival according to the transcript tumour expression did not show stadistical differences.

According to our data, *MDR3* and *MRP1* were more highly expressed in high-risk and in blastemal subtype WT. The next step was to correlate these results with the clinical parameters of patients (Series 1). Previous to this analysis, we confirmed that our patient sample series was representative. We analyzed by Kaplan-Meier the relation between disease-free survival (a clinical prognosis marker) with tumour subtype and risk. We confirmed that patients with blastemal subtype or with high-risk tumours had a reduced disease-free survival (Figure 4A and 4B) and were significantly more likely to relapse. These results confirm that our series represents what has been previously described in other published studies. Blastemal predominant and high-risk WT is more aggressive than other subtypes and has a poor outcome [16–17]. Next, we analyzed disease-free survival versus *MDR3* and *MRP1* transcript expression. We used a median cut off because it is more representative and less biased. Our results showed that WT patients with high transcript levels of *MDR3* had a significantly poorer prognosis than WT patients with low mRNA levels (Figure 4C). We performed an analogous analysis with *MRP1* but it did not show any statistical differences between expression levels (Figure 4D). In conclusion, *MDR3* and *MPR1* overexpression could be important biomarkers in high-risk WT but only the high expression of *MDR3* confers poor prognosis in treated patients.

### The level of MDR3 expression can be used to stratify the Wilms tumour blastemal subtype

**Figure 5:**
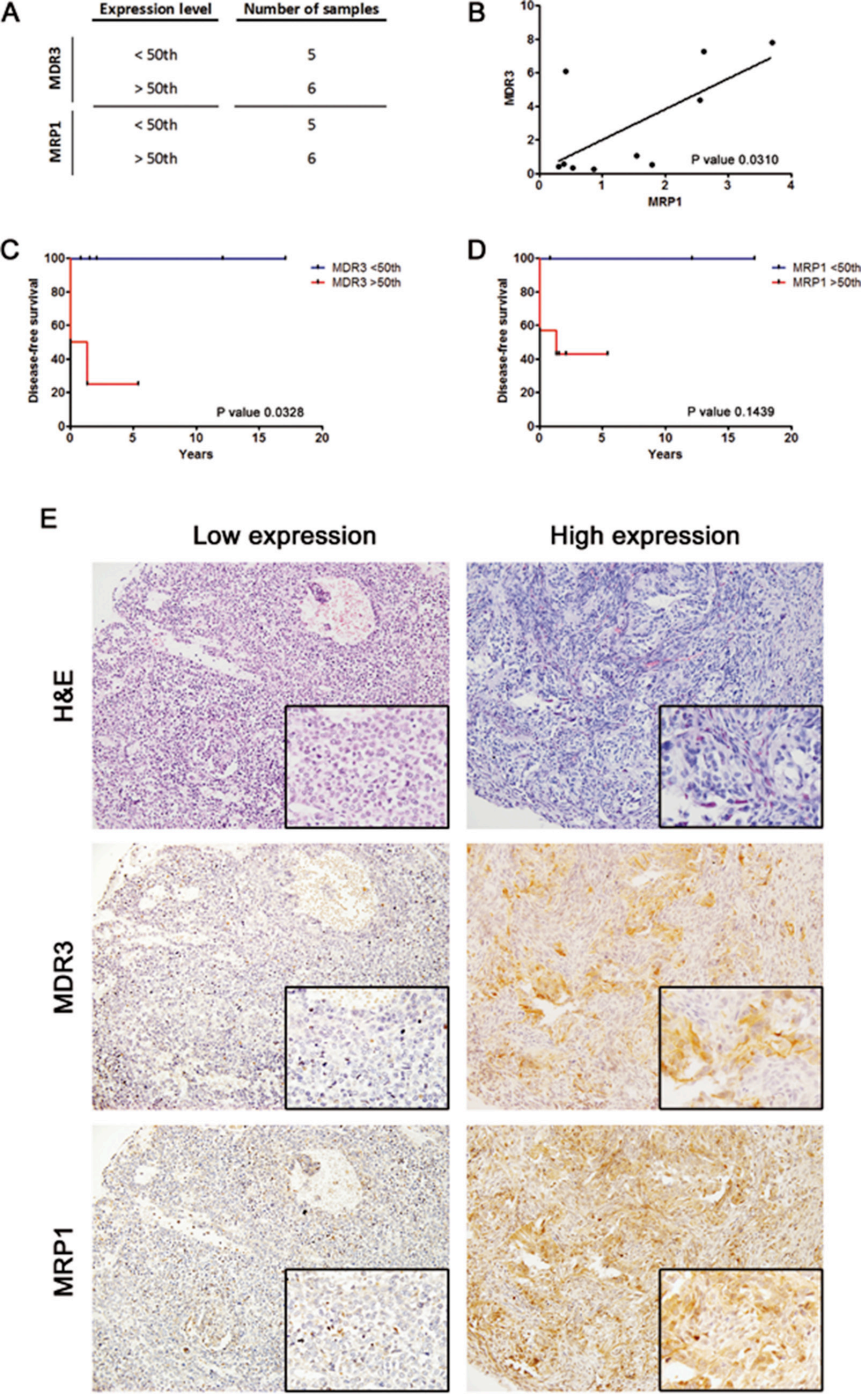
*MDR3* gene expression is associated with disease-free survival in the blastemal WT subtype and allows the stratification of patients (**A**) Distribution of the number of samples with high expression versus non/low *MDR3* and *MRP1* expression, respectively. The cutoff value corresponds to the median expression level of each gene in WT. (**B**) Correlation study of the expression of *MRP1* and *MDR3* in blastemal tumour samples. (**C**) *MDR3* Kaplan-Meier plot with disease-free survival according to the transcript blastemal tumour expression showed statistically significant differences between low and high expression. No patients with non/low expression *MDR3* relapsed. (**D**) *MRP1* Kaplan-Meier disease-free survival according to the transcript blastemal tumour expression did not show statistically significant differences. (**E**) Immunohistochemical detection of MDR3 and MRP1 in blastemal WT patient samples from tissue microarray (20× and 40× amplification).

The WT subtype with the worse clinical outcome is blastemal predominant. Applying the same cut off as in Figure 4C–4D, the number of blastemal WT samples in Series 1 with low or high expression levels of *MDR3* was 5 and 6 respectively, with similar numbers for *MRP1* expression (Figure 5A). From the Series 1 samples we obtained a positive and significant correlation between *MDR3* and *MRP1* transcript expression in blastemal tumour samples only (Figure 5B). Similar to the correlation previously identified in Figure 2D for all samples, blastemal WT with high *MDR3* expression also showed high levels of *MRP1*.

Surprisingly, we found blastemal WT samples with both high and low expression of *MDR3* and *MRP1*. Therefore, we were interested to examine whether the differences in expression had any clinical relevance. We analyzed by Kaplan-Meier the relation between disease-free survival and transcript expression levels. We observed that patients with high *MDR3* expression showed a higher relapse rate than patients with low *MDR3* expression (*p* = 0.0328). In fact, the subgroup with low expression did not relapse (Figure 5C). However, we could not confirm the relation between the progression of patients with blastemal predominant tumours and *MRP1* expression levels statistically due to the small sample size (Figure 5D). To confirm MDR3 and MRP1 as possible clinical markers for blastemal subtype stratification, further to the analysis of gene expression by qRT-PCR, we checked whether we could stratify WT subtype cases by the level of IHC labeling. There were only four blastemal cases in our TMA (Series 2) thus we were unable to do a statistical comparison. Nevertheless, we confirmed by IHC that there were different MDR3 and MRP1 expression levels in blastemal WT samples. We identified two types of blastemal WT, i) those with low MDR3 and MRP1 expression, and ii) those with high expression of both proteins (Figure 5E).

## DISCUSSION

Predicting the clinical behavior and prognosis of Wilms tumours can be difficult. Currently, the prediction of treatment outcome – whether the WT patient will respond to chemotherapy or not – is mainly based on the histopathology and stage of disease at the time of resection [34]. The aim of this study was to identify novel molecular prognostic markers by characterization of the expression profiles of multidrug resistant genes in WT samples. The role of multidrug resistant transport-associated proteins in chemotherapy-acquired drug resistance and its correlation with the evolution of the disease has not been fully investigated for WT. Our results showed a differential expression pattern of specific multidrug resistance genes after the treatment of WT patients. The majority of multidrug resistance transporters studied was downregulated or unchanged between tumours and control tissues. However *BCRP1, MDR3* and *MRP1* were upregulated in tumours versus control tissues. Among the multidrug resistant transporters studied, only MDR3 and MRP1 showed a significant correlation with clinicopathological parameters. Specifically, our retrospective results showed an increase in high-risk and blastemal predominant subtype tumours associated with MDR3 and MRP1 expression. There was a positive correlation between *MDR3* and *MRP1* expression in tumours but not in normal kidney. We suppose that the increased expression of the two transporters would confer more resistance to treatment in WT, as has recently been demonstrated in ovarian cancer and in several different cancer cell lines [35–36].

Confirming our data, many studies have highlighted the importance of MDR3 or MRP1 in malignant disease. A study of chronic lymphocytic leukemia reported the increased expression of MDR3 in patients with advanced disease in comparison to early stages [37–38]. Furthermore, in pediatric soft tissue sarcomas after chemotherapy, an increase in *MDR3* gene expression was described as well as in cholangiocarcinoma and cirrhosis [39]. For *MRP1*, high levels of the gene were associated with poor prognosis and high histological grade in childhood neuroblastoma [40–42], soft tissue sarcoma [43], and non-small cell lung cancer [44–46], and with recurrence in breast cancer patients who were treated with chemotherapy [47]. To our knowledge, only one previously reported study suggested the existence of a common expressing regulatory mechanism between MDR3 and MRP1 in high-risk tumours such as breast cancers and nephroblastoma [48].

Although *MDR3* and *MRP1* were overexpressed in high-risk WT and we found a positive correlation between them, only *MDR3* expression was negatively correlated with disease-free survival. In colorectal cancer, the disease free interval of patients treated by adjuvant chemotherapy was significantly shorter in patients with low transcript levels of *MDR3* [49]. In addition, the overall survival of liver cancer patients tended to be longer in those patients with high *MDR3* and *MRP1* expression compared to the control group [50]. Our results did not show any relation between *MRP1* and disease-free survival, but other studies demonstrated an association with patient survival in nephroblastoma [29] and with shorter time of disease progression in breast cancer [51]. The discrepancy in results obtained in different tumours may be due to different factors such as epigenetics, microenvironment, or compensation between different multidrug resistance proteins and altered tumour homeostasis versus healthy organs by inhibiting or overexpressing different multidrug resistance genes.

Predicting the clinical response of WT patients to chemotherapy treatment with current clinical tools can be difficult; therefore, there is a real need to identify molecular prognostic markers. The blastemal subtype of nephroblastoma after pre-operative chemotherapy is strongly associated with adverse outcome [52]. Our results showed that MDR3 could be a prognostic marker in blastemal and high-risk WT. Surprisingly, we observed two different blastemal WT patients defined by MDR3 and MRP1 expression, and relapse was associated with high MDR3 expression in blastemal WT. Results in MRP1 showed the same behavior but the number of samples was not high enough to reach statistical significance. Nevertheless, we observed a positive correlation between MDR3 and MRP1 in blastemal WT, stratifying blastemal WT into two groups with different clinical outcomes and confirming the importance of the multidrug resistance transports in blastemal WT. In accordance with our data, Barroca suggested that two types of blastema may occur in WT and that each of these types exhibit different chemotherapeutic sensitivities, proliferative properties, and abilities to undergo apoptosis or necrosis [53]. To date, the molecular effects of therapy on WT and the factors that produce chemoresistance in this tumour are not well understood. The identification of new prognostic/response factors for WT would enable the stratification of patients for optimal clinical strategy. Therefore, biomarkers like MDR3 could provide oncologists with a novel tool to identify patients with a high risk of recurrence in order to apply an appropriate therapy.

For an effective treatment design for WT patients, the role of multidrug resistance proteins must be considered. The role of transporters has been described as inducers of chemoresistance in several studies. In paclitaxel-, doxorubicin- and vincristine-resistant cell lines (colon and ovarian cancer cell lines), an increased expression of several drug resistance genes was identified [35]. Also, in meduloblastoma cell lines, the inhibition of ABC transporters (MDR3 and others) increased the efficacy of radiation therapy [54]. Resistance to chemotherapy may be due to high expression of multidrug resistance mechanisms already present in tumor cells before treatment, and/or it is also possible that chemotherapy modulates the expression of multidrug resistance proteins reducing treatment efficacy. Several studies showing that expression of MRP1 and MDR3, among others multidrug resistance genes may induce or increase after chemotherapy in pediatric tumors, soft tissue sarcomas and malignant melanoma [33, 48, 55]. In the SIOP-93-01 protocol, vincristine and actinomycin D treatment were given to the patient with localized tumor, and doxorubicin was included in patients with metastatic disease. It was known that MDR3 and MRP1 confer resistance to a variety of drugs like vincristine, actinomycin D and doxorubicin [30, 56–58]. In addition among these drugs, only, vincristine had been described as a MRP1 substrate [25, 59]. Our results suggested that the increased of MDR3 and MRP1 expression in high risk and blastemal WT conferred resistance to first line WT chemotherapy treatment. We hypothesize that this tumors had more active the efflux pumps and decreased the intracellular accumulation of this drugs due to the high expression of MDR3 and MPR1.

It will be interesting to explore the inhibition of transporters as an adjuvant treatment to the standard treatment of WT patients. Several studies have demonstrated that inhibition of multidrug resistance proteins improves the results of treatment-resistant tumours. For example, ovarian cancer cells inhibited by siRNA against MDR3 showed reversed resistance to paclitaxel [54, 60]. In neuroblastoma cells, an MRP1 inhibitor increased reversal of the therapeutic index of chemotherapy in mouse models [61]. Currently, several drugs associated with drug-induced liver injury (DILI), such as chlorpromazine, imipramine, itraconazole and haloperidol, are being tested as MDR3 inhibitors. However, these inhibitors are associated with undesirable side effects like liver damage [62]. The development of more specific peptidomimetic inhibitors, glutathione-conjugate analogs (MRP1 inhibitor), could prevent these adverse outcomes [63].

In summary, our findings suggest that MDR3 and MRP1 could have an important role in drug resistance in WT treatment. In addition, we have described a possible new biomarker for stratification of the WT blastemal subtype. We demonstrated that low expression of MDR3 in the blastemal subtype is associated with a good prognosis in this high-risk tumour. In the future, it will be interesting to perform prospective studies with larger number of samples to confirm MDR3 expression as a clinical biomarker for WT prognosis and treatment outcome.

## MATERIALS AND METHODS

### Patients and clinical samples

This retrospective study included two different sample series obtained between 1993 and 2006. Series 1 comprised 46 frozen tumors with 40 normal kidneys (no tumoral kidneys). In 40 cases, normal kidney and tumor paired samples could be obtained. From 6 patients there was only tumoral tissue without normal kidney available. Series 2 comprised a Tissue Microarray (TMA) with 31 paraffin-embedded samples. This series consisted of 9 paired tumor and normal kidney, and 22 tumoral samples with no matched non tumoral kidney. The control group (normal kidney) consisted of renal tissue from the non tumoral part of the resected specimen following tumor nephrectomy. Pathologists selected the farthest region to the tumor. All samples were analyzed by two experienced pathologists, who confirmed that the tumor samples were correctly identified as WT and the control kidneys did not present any morphological alteration. Histological stratification of the samples was performed according to SIOP [14] classification and the tissue samples were obtained from the Department of Pathology at the Hospital Universitario Virgen del Rocío (Seville, Spain). Approval of the Ethics Committee of this institution was obtained and written informed consent was obtained before registration of the patients from the HUVR-IBiS Biobank. The patient characteristics are summarized in Table 1.

**Table 1:** Clinicopathological features of the tumor samples

	Series 1	Series 2
**Samples**	Frozen	Paraffin
**Number of samples**	46	31
**Age in years (range)**	6 months-5 years	6 months-9 years
**Stage**		
I	21	17
II	8	2
III	7	5
IV	4	2
Data not available	6	5
**Risk**		
Low	2	0
Intermediate	15	24
High	8	7
Data not available	21	–
**Subtype**		
Blastemal	11	4
Epithelial	8	5
Stromal	6	4
Others	21	18

### Patients treatment

All patient series were treated by neoadjuvant chemotherapy before nephrectomy according to the SIOP-93-01 protocol. Patients with localized disease were treated with vincristine and actinomycin D during 4 weeks and those with metastatic disease received vincristine, actinomycin D and doxorubicin during 6 weeks. SIOP preoperative treatment protocol is summarized in Table 2.

**Table 2:** SIOP 93-01 preoperative treatment protocol

	Dosages	Medication Administration	Treatment regimens (weeks)
**Localized Stage**			
Vincristine	1.5 mg/m^2^	Intravenous	1st and 3th
Actinomycin D	45 μg/kg	intravenous	1st to 4th
**Metastatic Stage**			
Vincristine	1.5 mg/m^2^	intravenous	1st -3th-5th
Actinomycin D	45 μg/kg	intravenous	1st to 6th
Doxorrubicin	50 mg/m^2^	intravenous	1st and 5th

### mRNA expression analysis

The expression of selected genes was analyzed by qRT-PCR. RNA was isolated from 88 frozen samples (40 kidneys and 46 WT) from which sufficient material was available with the miRVana miRNA Isolation Kit (Ambion; Life Technologies, NY, USA). The quantity and quality of the total RNA was determined with a Nanodrop ND-2000 Spectrophotometer (Thermo Scientific). Prior reverse transcription was performed using the TaqMan Reverse Transcription Kit (Applied Biosystems; Life Technologies) in the GeneAmp PCR 9700 system and qRT-PCR amplification with the TaqMan Universal PCR Master Mix (Applied Biosystems). All qRT-PCR measurements were obtained in a 7900HT Fast Real Time PCR System with the ExpressionSuite Software v1.0 (Applied Biosystems). Table 3 summarizes the Taqman probes utilized in this study.

**Table 3:** TaqMan Gene Expression probes employed for qRT-PCR

Genes	Brand	Reference	Amplicon Length
BCRP1	Applied Biosystems	Hs01053790_m1	83 bp
GAPDH	Applied Biosystems	Hs99999905_m1	93 bp
HRPT1	Operon	custom design	149bp
MDR1	Applied Biosystems	Hs00184500_m1	67 bp
MDR3	Applied Biosystems	Hs00240956_m1	73 bp
MRP1	Applied Biosystems	Hs01561502_m1	69 bp
MRP2	Applied Biosystems	Hs00166123_m1	75 bp
MRP3	Applied Biosystems	Hs00978473_m1	57 bp
MVP1	Applied Biosystems	Hs00245438_m1	65 bp
TEP1	Applied Biosystems	Hs00200091_m1	66 bp
TPT1	Applied Biosystems	Hs02621289_g1	131 bp
vPARP	Applied Biosystems	Hs00173105_m1	118 bp
**HRPT1**	Primers Sequence		
Forward	5′ - CAGCCCTGCCGTCGTCGTGATA - 3′		
Reverse	5′ - AGCAAGACGTTCAGTCCTGTC - 3′		

### Protein analysis

Proteins were extracted from kidney and WT frozen tissues in RIPA buffer (150 mM NaCl, 1% (v/v) NP40, 50 mM Tris-HCl pH 8.0, 0.1% (v/v) SDS, 1 mM EDTA and 0.5% (w/v) deoxycholate) supplemented with 10 mM NaF and 2 mM NaOv. Samples were incubated for 20 min on ice and centrifuged for 15 min at 13000 r.p.m. at 4°C. Supernatants were collected and quantified using the BCA Protein Assay Kit (Thermo Fisher Scientific Inc.). Equivalent amounts of proteins were resolved by SDS polyacrylamide gel electrophoresis, and transferred to polyvinylidene difluoride membranes (Immobilon-P, Millipore, Darmstadt, Germany). Immunoblotting was performed using the following antibodies: anti-ABCB4 (Abcam-ab184878) overnight at 1:200 dilution; anti-MRP1 (Abcam-ab137406) overnight at 1:200 dilution; anti-β-actin (A5441 clone AC-15) for 1.5 h at 1:10000 dilution; anti-rabbit IgG, HRP (Cell Signaling, ref#7074) for 1 h at 1:10000; anti-mouse IgG-HRP (Cell Signaling, ref#7076) for 1 h at 1:10000. Protein bands were visualized using the Clarity Western ECL Substrate chemiluminescence detection kit (Bio-Rad, ref#170-5060). All of the antibodies were previously analyzed for antigen specificity in our laboratory, all conditions being optimized for specific antigen detection, with elimination of nonspecific reactivity.

### TMA and Immunohistochemistry (IHC)

Tissue sections (5 μm) from formalin-fixed paraffin-embedded (FFPE) of WT were stained with hematoxylin and eosin. Representative malignant areas from 31 WT patients were carefully selected from the stained sections of each tumour, and two 1 mm diameter tissue cores were obtained from each sample to build up the TMA in duplicate. Five micron-thick TMA sections were dewaxed, rehydrated, and immersed in 3% H2O2 aqueous solution for 30 minutes to exhaust endogenous peroxidase. Heat-induced epitope retrieval was performed with 1 mM EDTA (pH 9.0) in a microwave oven. Sections were incubated overnight at 4°C with the primary antibodies anti-ABCB4 (MDR3) (Sigma HPA053288; overnight at 1:20 dilution) and Anti-MRP1 (Abcam-ab137406; overnight at 1:200 dilution). Peroxidase-labeled secondary antibodies and 3,3′-diaminobenzidine were applied according to manufacturer's protocol (EnVision, Dako). Slides were then counterstained with hematoxylin and mounted. Sections where the primary antibody was omitted were used as negative controls. The plasma membrane and cytoplasmic was stained for both transporters (MDR3 and MRP1). Immunostains were scored as negative/positive expression according to the stain intensity and proportion of stained cells. Only tumoural cells evaluated as clearly stained were considered to be positive. The IHC results were evaluated by two pathologists (E.A. and C.S.) who scored the average expression of markers in duplicate samples.

### Statistical analysis

Gene expression differences between control (kidney) and WT were evaluated with the Mann–Whitney *U*-test for two groups, and with the Kruskal–Wallis test for more than two groups, followed by Dunn's multiple comparison post test. Paired samples *t*-test was applied to compare the mean level of expression within the same specimens. The Spearman´s Rank test was used to quantify the correlation of expression between different genes. Fisher´s exact test was used to evaluate differences between immunohistochemical expression detection of MDR3 and MRP1. The disease-free survival time was analyzed using the Kaplan–Meier estimator and the Wilcoxon test. For all analyses, *P*-values of ≤ 0.05 were considered statistically significant. Analyses were performed using the Prism 4.0 software (GraphPad).
