# Correction: Multidrug resistance transporter profile reveals MDR3 as a marker for stratification of blastemal Wilms tumour patients

**DOI:** 10.18632/oncotarget.20708

**Published:** 2017-09-08

**Authors:** Lourdes Hontecillas-Prieto, Daniel J. Garcia-Dominguez, Diego Pascual Vaca, Rosa Garcia-Mejias, David Marcilla, Gema L. Ramirez-Villar, Carmen Saez, Enrique de Álava

**Present**: The affiliation information is incomplete.

**Correct**: Additional affiliation information is shown below. The publishers sincerely apologize for this oversight.

Original article: Oncotarget. 2017; 8:11173-11186. https://doi.org/10.18632/oncotarget.14491

**Lourdes Hontecillas-Prieto**^1,^*, **Daniel J. Garcia-Dominguez**^1,^*, **Diego Pascual Vaca**^2^, **Rosa Garcia-Mejias**^1^, **David Marcilla**^2^, **Gema L. Ramirez-Villar**^3^, **Carmen Saez**^1,2,^**, **Enrique de Álava**^1,2,^**

^1^ Institute of Biomedicine of Seville (IBiS), Hospital Universitario Virgen del Rocío/CSIC/Universidad de Sevilla, CIBERONC. Seville, Spain

^2^ Pathology Unit, Hospital Universitario Virgen del Rocío/CSIC/Universidad de Sevilla, CIBERONC. Seville, Spain

^3^ Pediatric Oncology Unit, Hospital Universitario Virgen del Rocío/CSIC/Universidad de Sevilla, Seville, Spain

* These authors contributed equally to this work as first authors

** These authors contributed equally to this work as senior authors

